# Associations between multimodal retinal measurements and cognitive functions in patients with cerebral small vessel disease

**DOI:** 10.3389/fnins.2026.1773266

**Published:** 2026-03-20

**Authors:** Meizi Wang, Lan Ma, Mengxi Zhao, Qianqian Yang, Yuetong Liu, Hongyi Yan, Yuan Gao, Ning He, Xuxiang Zhang, Ling Guan, Tingting Wang

**Affiliations:** 1 Department of Ophthalmology, Beijing Tiantan Hospital, Capital Medical University, Beijing, China; 2 Department of Neurology, Liangxiang Hospital, Beijing, China; 3 Department of Neurology, Beijing Tiantan Hospital, Capital Medical University, Beijing, China; 4 China National Clinical Research Center for Neurological Diseases, Beijing, China; 5 Department of Ophthalmology, Xuanwu Hospital, Capital Medical University, Beijing, China; 6 Engineering Research Center of Molecular and Neuro-Imaging, Ministry of Education, School of Life Science and Technology, Xidian University, Xi’an, China; 7 Advanced Research Institute of Multidisciplinary Sciences, Beijing Institute of Technology, Beijing, China; 8 Department of Medicine, The University of British Columbia, Vancouver, BC, Canada

**Keywords:** cerebral small vessel disease, cognitive dysfunction, multimodal retinal evaluation, optical coherence tomography angiography, retinal vessels

## Abstract

**Purpose:**

To investigate the associations between retinal parameters and cognitive impairment and identify retinal biomarkers for detecting cognitive dysfunction in patients with cerebral small vessel disease (CSVD).

**Methods:**

This cross-sectional study enrolled 125 patients with CSVD-related white matter hyperintensities. Participants were independent in daily activities and free of significant ophthalmic diseases. Cognition was assessed using the Mini-Mental State Examination (MMSE). Multimodal retinal imaging was employed to evaluate retinal structural and vascular features, including peripapillary retinal nerve fibre layer (pRNFL) and ganglion cell complex thicknesses and vessel densities (VDs) of the radial peripapillary capillary (RPC) network, macular superficial retinal capillary plexus (SRCP), and deep RCP (DRCP). The central retina vascular diameter, mean angle, angle tortuosity, arc length tortuosity, and fractal dimension of the retinal arteries and veins were quantified using a fully automated vessel segmentation and parameter calculation method. Linear and logistic regression models were applied to assess the associations between retinal parameters with general cognitive domains (general cognition, memory, visuospatial space, attention and numeracy, language, and orientation). Receiver operating characteristic (ROC) curves and areas under the ROC curve (AUCs) were used to evaluate the predictive performance of the retinal biomarkers.

**Results:**

A smaller central retinal arteriolar equivalent/central retinal venular equivalent ratio [odds ratio (OR) = 0.013, *p* = 0.035] and lower VDs in whole macular SRCP (OR = 0.864, *p* = 0.029), perifoveal SRCP (OR = 0.862, *p* = 0.027), perifoveal superior SRCP (OR = 0.855, *p* = 0.014), perifoveal nasal SRCP (OR = 0.833, *p* = 0.008), and inside-disc capillary RPC network (OR = 0.901, *p* = 0.028) were significantly associated with lower MMSE scores. Attention and orientation scores were significantly correlated with the VDs of the SRCP (*β* = 0.734, *p* = 0.027 and β = −9.460, *p* = 0.037, respectively) and DRCP (*β* = −0.553, *p* = 0.004 and *p* = 0.044, respectively), whereas visuospatial and language function scores were associated with the VD of the inside-disc RPC network (*β* = 0.018, *p* = 0.026 and β = 0.068, *p* = 0.012, respectively). The VDs of the whole macular, perifoveal, perifoveal superior, and nasal SRCPs achieved AUCs of 0.71–0.74 for screening cognitive impairment.

**Conclusion:**

Specific retina parameters are associated with cognitive decline in patients with CSVD. These findings suggest that multimodal retinal evaluation might provide an objective, imaging-based adjunct to conventional subjective cognitive tests for screening cognitive impairment in patients with CSVD.

**Clinical trial registration:**

https://www.chictr.org.cn/, identifier ChiCTR 2100043346.

## Introduction

Cerebral small vessel disease (CSVD) comprises a group of disorders that affect the cerebral microcirculation (Cannistraro et al., 2019). It is a major contributor to cognitive impairment, and it has been implicated in more than 50% of Alzheimer’s disease (AD) cases (Li et al., 2018; Wardlaw et al., 2019). The retina and brain share similar anatomic, embryologic, and physiologic characteristics (Patton et al., 2005). Because the retina is directly accessible and not obscured by the skull, it provides a unique, non-invasive window into the cerebral microvascular environment (London et al., 2013). This connection is further supported by prior genetic studies that identified shared genetic risk variants between retinal vascular traits and CSVD, reinforcing the notion that the retinal microvasculature might reflect genetically influenced cerebrovascular integrity (Lu et al., 2023). Previous investigations demonstrated that reduced retinal vascular branching is associated with white matter damage and decreased cerebrovascular reactivity in patients with CSVD (Wiseman et al., 2023).

Currently, the assessment of cognitive impairment primarily depends on subjective scales such as the Mini-Mental State Examination (MMSE) and Montreal Cognitive Assessment (MoCA) scales, the accuracy of which can vary with examiners’ experience. Therefore, developing non-invasive, objective approaches to evaluate cognitive impairment in patients with CSVD is of great importance. Emerging evidence has suggested that structural alterations in the retina mirror the neuropathological changes underlying cognitive impairment, and they could help differentiate dementia subtypes (McGrory et al., 2017; Min et al., 2025). Recent work indicates that specific retinal changes can reflect distinct molecular pathological processes, such as amyloid deposition and neuroinflammation, providing a potential pathway for the *in vivo* detection of early neurodegenerative pathology (Tian et al., 2023). Specifically, in preclinical models such as the *APOE4* knockout AD mouse model, retinal structural and microvascular alterations coincide with cognitive decline, supporting the rationale for investigating retinal biomarkers in human CSVD, a condition in which cerebral microvascular dysfunction often precedes or accompanies cognitive impairment (Abhyankar et al., 2025).

Optical coherence tomography (OCT) and optical coherence tomography angiography (OCTA) are advanced, non-invasive imaging techniques that provide high-resolution visualisation of the retinal structure and microvasculature *in vivo*. These modalities permit the objective quantification of retinal perfusion and enable early detection of microvascular changes that might not yet be detectable by conventional clinical or radiological assessments (Wiseman et al., 2023). Recent studies have revealed that reduced vessel densities (VDs) in the temporal quadrant of the radial peripapillary capillary (RPC) network (Lee et al., 2020), macular superficial retinal capillary plexus (SRCP) (Wang et al., 2021), and macular deep retinal capillary plexus (DRCP) (Huang et al., 2024) are associated with cognitive impairment in patients with CSVD. Furthermore, thinning of the peripapillary retinal nerve fibre layer (pRNFL) has been closely linked to CSVD, indicating a possible specific neuroretinal biomarker (Ito et al., 2020).

Although previous studies reported associations between isolated retinal parameters and cognitive decline in CSVD, most focused on single modalities or limited vascular regions. To our knowledge, this is among the first studies in CSVD to comprehensively integrate multimodal retinal imaging, including structural OCT, OCTA-based vessel density, and automated fundus-based vascular morphology analysis, within the same cohort. This approach permits a more holistic assessment of retinal microvascular and structural integrity, and enables the identification of specific, potentially synergistic biomarkers associated with distinct cognitive domains. In this manner, we aim to provide a more refined, clinically applicable framework for the objective screening of cognitive impairment in CSVD.

## Methods

This study was approved by the Ethics Committee of Beijing Tiantan Hospital (ethical approval number KY2019-140-02) and performed in accordance with the principles of the Declaration of Helsinki. The registration number is ChiCTR 2100043346. Informed written consent was obtained either from the participants or from their legal representatives.

## Study design and population

This cross-sectional study was conducted as part of a national hospital-based cohort of patients with CSVD. Patients diagnosed with CSVD-related white matter hyperintensities (WMHs) at Beijing Tiantan Hospital from January 2020 to November 2023 were enrolled.

Individuals with significant ophthalmic diseases that could confound retinal imaging or cognitive assessment, including glaucoma, age-related macular degeneration, diabetic retinopathy, severe cataracts (Lens Opacities Classification System III grade ≥3), retinal vascular occlusions, uveitis, and high myopia (spherical equivalent ≤ −6.0 D), were excluded. Other exclusion criteria included a history of intraocular surgery and an inability to maintain a sitting position or fixate steadily for 10–20 s. The detailed inclusion and exclusion criteria and the process of assessment are listed in Supplementary Table 1.

## Study assessment

### Assessment of demographic and clinical information

Demographic and clinical information, including age, sex, education, medical history of hypertension, diabetes mellitus, and hyperlipidaemia, and smoking and drinking status, was recorded from patients’ medical records or self-reports.

### Neuroimaging examinations

Brain magnetic resonance imaging (MRI) was performed using a 3.0 T scanner (Siemens MAGNETOM Prisma, Erlangen, Germany) equipped with a Siemens 64-channel Prisma head coil. The MRI sequences included axial T1-weighted imaging, axial T2-weighted imaging, axial fluid-attenuated inversion-recovery, diffusion-weighted imaging, and susceptibility-weighted imaging. Imaging markers of CSVD, including recent subcortical small infarctions, WMHs, lacunes, periventricular spaces, and cerebral microbleeds, were defined according to the Standards for Reporting Vascular Changes on Euroimaging (STRIVE) guidelines (Wardlaw et al., 2013). The CSVD total burden score, which ranges from 0 to 4, was calculated according to the number of different STRIVE signs (Staals et al., 2014). CSVD imaging markers were assessed by two trained physicians (ZMX and ML) who were blinded to the patient information. Discrepancies were resolved by an experienced neurologist (WTT).

### Multimodal retinal measurements

All patients underwent multimodal retinal measurements, including assessments of retinal structural features and vascular morphology and density.

### Retina structure assessments

Retinal structural features, including the thicknesses of the pRNFL and ganglion cell complex (GCC), were examined using a spectral-domain OCT system (RTVue XR Avanti with AngioVue software; Optovue, Inc., Fremont, CA, USA). The pRNFL thickness was derived from a high-density circular scan protocol (3.45 mm diameter centred on the optic nerve head) using the device’s standard ONH (optic nerve head) cube scan mode, which acquires 200 × 200 A-scans across a 6 × 6 mm^2^ area, from which a 3.45-mm-diameter circular pRNFL measurement is automatically extracted by the built-in software. For GCC analysis, a macular cube scan covering a 6 × 6 mm^2^ area centred on the fovea was acquired. The scan density was 200 × 200 A-scans (200 B-scans × 200 A-scans per B-scan), with two-frame averaging per B-scan. The GCC thickness was automatically calculated by the software as the combined thickness of the retinal nerve fibre layer (RNFL), ganglion cell layer, and inner plexiform layer within an elliptical annulus centred on the fovea.

### Retina vascular assessment

Retinal vascular density features were assessed *via* OCTA (RTVue XR Avanti with AngioVue software; Optovue, Inc.). All OCTA scans were reviewed by an operator blinded to clinical and cognitive data for quality assessment. Scans with signal strength index < 60, significant motion artefacts, segmentation errors, or projection artefacts were either corrected using built-in software or excluded from analysis. The AngioVue disc mode was used to assess the RPC in the papillary network within a 4.5 × 4.5 mm^2^ area. Specifically, the RPC network, inside-disc RPC network (the RPCs in an area inside an ellipse fitted to the optic disc boundary), and peripapillary RPC network (the RPCs within a 0.75-mm-wide elliptical annulus extending outwards from the optic disc boundary) were measured. The capillary VDs of the RPC network were calculated accordingly (Rao et al., 2016). The patients underwent a macular OCTA scan using the Angio Retina protocol (scan area: 6 × 6 mm^2^) with a scan density of 304 × 304 A-scans (304 B-scans, each B-scan comprising 304 A-scans). This protocol is termed the “high-density” mode on the Optovue platform. Each B-scan was repeated twice at the same location for motion correction and averaging. Its VD was automatically analysed in two different retinal vascular networks: the SRCP (located between the internal limiting membrane and 10 μm above the inner plexiform layer) and DRCP (located between 10 μm above the inner plexiform layer and 10 μm below the outer plexiform layers). The macular region was divided into three concentric subfields according to the Early Treatment Diabetic Retinopathy Study grid: the foveal (central 1-mm-diameter circle), parafoveal (1–3 mm annular ring), and perifoveal (3–6 mm annular ring) subfields. The para- and perifoveal regions were further subdivided into four quadrants: superior, inferior, nasal, and temporal. Moreover, the acircularity index, the area density of the foveal avascular zone (FAZ), and the foveal vessel density within a 300-μm-wide zone surrounding the FAZ (FD-300) (Inanc et al., 2019) were analysed. Details of these regions are illustrated in Supplementary Figures 1–3.

The retinal vascular morphology was assessed by fundus photography (Nonmyd WX, Kowa Inc., Tokyo, Japan) using a fully automatic retinal vessel segmentation and parameter calculation system, as detailed previously (China Patent Application No: CN202211272854.6). All images were analysed using the same automated segmentation and calculation system. These methods included a deep learning algorithm for segmenting the retinal arteries and veins and respective mathematical formulas for calculating the retinal arteriovenous parameters based on the segmented vessels. These parameters included the mean angle, angle tortuosity, arc length tortuosity, and fractal dimension of the retinal arteries and veins. The central retinal arteriolar equivalent (CRAE), central retinal venular equivalent (CRVE), and CRAE/CRVE ratio were also derived. Details of the analysis are presented in Supplementary Figure 4.

### Other retinal assessments

In addition, the best-corrected visual acuity, refractive error, intraocular pressure (Canon TX-20P; Canon, Tokyo, Japan), and a slit lamp were used to assess ocular conditions of the patients to exclude ophthalmic diseases.

### Cognitive assessment

Cognitive function was assessed using the MMSE (Rosselli et al., 2006). The total MMSE score is 30, comprising six points for memory (immediate and delayed recall), one point for visuospatial ability (two-dimensional figure copying), five points for attention and calculation, eight points for language, and 10 points for orientation. Higher scores indicate better cognitive performance. MMSE score < 24 was considered to indicate dementia (Petersen et al., 1999). In this study, the MMSE score was analysed as a continuous or categorical variable. All tests were performed by two professionally trained researchers (LYT and YQQ).

### Statistical analysis

For each participant, we included the eye with better parameter quality in the analysis. Categorical variables are presented as percentages, and continuous variables are presented as the mean and standard deviation or median and interquartile range according to the data distribution. Comparisons of patient characteristics and retina parameters between the normal and dementia groups were conducted using the chi-squared test and Fisher’s exact test for categorical variables. For continuous variables, if the data met the assumptions of normality and homogeneity of variance, an independent-samples *t*-test was used to compare the two groups; otherwise, the Mann–Whitney U test was applied. Associations between retinal parameters and global cognitive function were assessed using univariate and multivariable logistic regression. Only parameters displaying significant group differences according to a t-test/Mann–Whitney U test were entered into regression analyses. Associations between retinal parameters and domain-specific cognitive function were assessed using multivariable liner regression. In the multivariable analyses, we adjusted for prespecified clinical confounders: age, hypertension, diabetes, ischaemic stroke, Fazekas WMH scores, and CSVD burden score. To further account for potential confounding related to cognitive reserve, education level was additionally included as a covariate in a secondary model to evaluate the robustness of the associations. Results are presented as odds ratios (OR) with 95% confidence intervals (CIs) for logistic models and beta coefficients (*β*) with 95% CIs for linear models. The screening performance of retinal features for cognitive function was estimated by receiver operating characteristic (ROC) curves and the area under the curve (AUC).

Statistical significance was set at *p* < 0.05. All statistical analyses were conducted using SAS software (version 9.4, SAS, Cary, NC, USA).

## Results

### Patient characteristics

**Figure 1:**
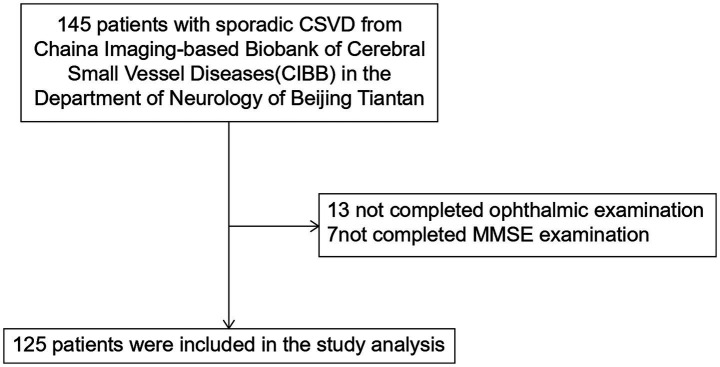
The flowchart of the study.

We screened 145 patients with CSVD at Beijing Tiantan Hospital from January 2020 to November 2023. Among them, 7 patients who did not complete the MMSE assessment and 13 patients who failed to complete the ophthalmological examinations were excluded. Consequently, 125 patients (mean age: 60.44 ± 10.56 years; 56.8% male) were included in the final analysis. The mean and median MMSE scores were 24.22 and 25, respectively. In total, 40 patients met the criteria for dementia, whereas 85 patients were classified as without dementia. The flowchart of the study is presented in Figure 1.

The demographic and clinical characteristics of the patients are summarised in Table 1. Compared with patients with normal cognitive function, those with dementia were older (*p* = 0.002), they had a higher prevalence of diabetes (*p* < 0.001) and ischaemic stroke (*p* = 0.013), and they had higher Fazekas scores for both periventricular (*p* = 0.006) and deep WMHs (*p =* 0.003). The overall CSVD burden score was also significantly greater in the dementia group (*p* < 0.001). To mitigate potential magnification bias, we excluded participants with high refractive error (spherical equivalent beyond ±6.0 D) and verified that the included cohort had mild refractive error on average (Table 1).

**Table 1:** Demographics and clinical information of patients with cerebral small vessel disease (CSVD).

Characteristics	Patients with CSVD (*n* = 125)			
	Total patients (*n* = 125)	Patients without dementia (*n* = 85)	Patients with dementia (*n* = 40)	*p*
Age (years)	60.44 ± 10.56	58.51 ± 10.07	64.58 ± 10.50	0.002
Sex (male/female)	71/54	45/40	26/14	0.204
Education (%)				0.052
Undergraduate/college degree or above	30 (24.0%)	26 (30.6%)	4 (10%)	
Senior high school	35 (28.0%)	24 (28.2%)	11 (27.5%)	
Junior high school	42 (33.6%)	27 (31.8%)	15 (37.5%)	
Primary school	12 (9.6%)	6 (7.1%)	6 (15%)	
Illiteracy	4 (3.2%)	0 (0%)	4 (10%)	
Medical history (%)				
Hypertension	83/42	53/32	30/10	0.163
Diabetes	31/94	13/72	18/22	<0.001
Coronary heart disease	16/109	8/77	8/32	0.098
Ischemic stroke	49/76	27/58	22/18	0.013
Smoking	45/80	30/55	15/25	0.811
Alcohol drinking	46/79	32/53	14/26	0.775
BMI (kg/m^2^)	25.69 ± 3.37	25.86 ± 3.46	24.89 ± 2.89	0.145
‌Fazekas score-PWMH [mean (IQR)]	2 (2–3)	2 (1–3)	3 (2–3)	0.006
‌Fazekas score-DWMH [mean (IQR)]	2 (1–3)	2 (1–3)	3 (2–3)	0.003
CSVD burden score [mean (IQR)]	3 (2–4)	3 (1–4)	3 (3–4)	0.000
IOP (mmHg)	14.00 ± 2.45	14.04 ± 2.52	13.91 ± 2.33	0.734
Refractive error	−0.16 ± 1.11	−0.18 ± 1.12	−0.11 ± 1.11	0.778

MMSE, Mini-Mental State Examination; BMI, body mass index; WMH, white matter hyperintensity; PWMH, perivascular white matter hyperintensity; DWMH, deep white matter hyperintensity; SD, standard deviation; IQR, interquartile range. The CSVD burden score was calculated by counting different Standards for Reporting Vascular Changes on Euroimaging signs. IOP, intraocular pressure.

### Associations between retinal features and overall cognitive function in patients with CSVD

Compared with the findings in patients without dementia, those with dementia exhibited significantly lower VDs in the whole macular SRCP and across all para- and perifoveal SRCP subfields. They also displayed reduced VDs in the whole macular DRCP and all corresponding subfields, as well as decreases in the FD-300 area density, inside-disc RPC and inside-disc capillary RPC VDs, artery mean angle, and CRAE/CRVE ratio (all *p* < 0.05, Table 2).

**Table 2:** Comparison of retinal parameters between patients with CSVD with and without dementia.

Retinal parameters, mean ± SD	Patients with CSVD (*n* = 125)			
	Total patients (*n* = 125)	Patients without dementia (*n* = 85)	Patients with dementia (*n* = 40)	*p*
BCVA (LogMAR)	0.06 ± 0.09	0.05 ± 0.08	0.10 ± 0.09	0.018
Average pRNFL thickness	100.28 ± 10.01	100.73 ± 9.89	101.20 ± 12.66	0.482
GCC	95.78 ± 7.25	96.30 ± 7.03	94.73 ± 7.66	0.265
Whole macular SRCP	50.15 ± 4.03	51.05 ± 3.63	48.19 ± 4.21	0.000
Foveal SRCP	17.59 ± 6.75	17.25 ± 7.21	18.33 ± 5.63	0.428
Parafoveal SRCP	51.79 ± 5.51	52.90 ± 5.28	49.35 ± 5.69	0.000
Parafoveal temporal SRCP	52.15 ± 5.23	53.14 ± 4.88	49.97 ± 5.37	0.001
Parafoveal superior SRCP	52.13 ± 6.88	53.31 ± 6.74	49.54 ± 6.56	0.001
Parafoveal nasal SRCP	50.54 ± 6.30	51.65 ± 6.00	48.09 ± 6.33	0.001
Parafoveal inferior SRCP	52.32 ± 5.60	53.48 ± 4.75	49.77 ± 6.49	0.002
Perifoveal SRCP	50.73 ± 4.12	51.67 ± 3.65	48.66 ± 4.38	0.000
Perifoveal temporal SRCP	47.13 ± 4.67	47.99 ± 4.16	45.25 ± 5.20	0.004
Perifoveal superior SRCP	51.00 ± 4.54	52.10 ± 3.86	48.57 ± 5.02	0.000
Perifoveal nasal SRCP	54.13 ± 3.97	55.16 ± 3.2	51.89 ± 4.02	0.000
Perifoveal inferior SRCP	50.59 ± 4.36	51.40 ± 4.09	48.80 ± 4.45	0.002
Whole macular DRCP	51.25 ± 5.38	52.33 ± 5.09	48.89 ± 5.31	0.002
Foveal DRCP	32.60 ± 7.96	32.65 ± 8.61	32.51 ± 6.41	0.701
Parafoveal DRCP	54.99 ± 4.44	55.52 ± 4.59	53.83 ± 3.90	0.012
Parafoveal temporal DRCP	56.10 ± 4.39	55.65 ± 4.32	54.88 ± 4.38	0.006
Parafoveal superior DRCP	54.34 ± 6.29	54.89 ± 6.78	53.14 ± 4.94	0.014
Parafoveal nasal DRCP	55.42 ± 4.99	55.77 ± 5.46	54.66 ± 3.71	0.024
Parafoveal inferior DRCP	54.09 ± 4.40	54.75 ± 4.14	52.62 ± 4.65	0.021
Perifoveal DRCP	52.62 ± 6.04	53.93 ± 5.51	49.77 ± 6.24	0.001
Perifoveal temporal DRCP	55.00 ± 5.24	56.07 ± 4.70	52.66 ± 5.66	0.002
Perifoveal superior DRCP	52.48 ± 6.58	53.91 ± 5.84	49.33 ± 7.07	0.001
Perifoveal nasal DRCP	51.16 ± 6.74	52.36 ± 6.39	48.54 ± 6.83	0.004
Perifoveal inferior DRCP	51.88 ± 6.79	53.38 ± 6.44	48.63 ± 6.74	0.001
FAZ area	0.34 ± 0.12	0.34 ± 0.12	0.33 ± 0.12	0.416
Acircularity index	1.11 ± 0.04	1.11 ± 0.04	1.11 ± 0.05	0.548
FD-300 area density	53.53 ± 4.95	54.48 ± 4.62	51.48 ± 5.06	0.003
Whole RPC image	56.05 ± 2.87	56.46 ± 2.16	55.21 ± 3.85	0.354
Capillary RPC	49.53 ± 2.81	49.83 ± 2.25	48.91 ± 3.67	0.652
Inside-disc RPC	59.82 ± 4.72	60.42 ± 4.10	57.59 ± 6.34	0.044
Inside-Disc capillary RPC	49.86 ± 5.67	50.63 ± 4.95	47.00 ± 7.20	0.034
Peripapillary RPC	58.27 ± 3.21	58.76 ± 2.34	57.25 ± 4.33	0.344
Peripapillary–capillary RPC	51.96 ± 3.18	52.30 ± 2.54	51.25 ± 4.16	0.595
Artery mean angle	62.32 ± 10.91	64.13 ± 8.72	53.58 ± 13.88	0.037
Artery angle tortuosity	1.47 ± 0.05	1.47 ± 0.05	1.47 ± 0.06	0.812
Artery arc length tortuosity	1.08 ± 0.02	1.09 ± 0.02	1.09 ± 0.02	0.686
Artery fractal dimension	2.51 ± 0.16	2.53 ± 0.14	2.46 ± 0.18	0.050
CRAE	137.00 ± 29,39	141.23 ± 24.75	127.20 ± 36.59	0.052
CRVE	210.05 ± 24.31	207.40 ± 23.40	215.47 ± 25.52.64	0.085
CRAE/CRVE	0.66 ± 0.15	0.69 ± 0.14	0.60 ± 0.14	0.004
Vein mean angle	65.52 ± 12.00	65.57 ± 10.65	65.42 ± 14.59	0.527
Vein angle tortuosity	1.45 ± 0.06	1.45 ± 0.05	1.45 ± 0.06	0.096
Vein arc length tortuosity	1.09 ± 0.02	1.09 ± 0.02	1.09 ± 0.02	0.752
Vein fractal dimension	2.63 ± 0.08	2.63 ± 0.09	2.63 ± 0.07	0.748

SD, standard deviation; MMSE, Mini-Mental State Examination; BCVA, best-corrected visual acuity; SRCP, superficial retinal capillary plexus; DRCP, deep retinal capillary plexus; CRVE, central retinal venular equivalent; CRAE, central retinal arteriolar equivalent; FAZ, foveal avascular zone; FD-300 area density, foveal vessel density in a 300-μm-wide zone around the FAZ; GCC, ganglion cell complex; pRNFL, peripapillary retinal nerve fibre layer; RPC, radial peripapillary capillary.

The results of univariate and multivariable regression analyses of retinal parameters are presented in Table 3. In univariate analysis, dementia was significantly associated with a smaller CRAE/CRVE ratio; reduced VDs in the whole macular SRCP, perifoveal SRCP, and all subfields of the SRCP; lower FD-300 area density, and decreased inside-disc RPC and capillary RPC densities (all *p* < 0.05). After adjusting for age; medical history of hypertension, diabetes, and ischaemic stroke; Fazekas WMH scores; and CSVD burden scores, several retinal parameters remained significantly associated with dementia, including a smaller CRAE/CRVE ratio (*p* = 0.035) and lower VDs of the whole macular SRCP (*p* = 0.029), parafoveal inferior SRCP (*p* = 0.032), perifoveal SRCP (*p* = 0.027), perifoveal superior SRCP (*p* = 0.014), perifoveal nasal SRCP (*p* = 0.008), and inside-disc capillary RPC network (*p* = 0.028). Furthermore, after adjusting for education, a smaller CRAE/CRVE ratio (*p* = 0.045) and lower VDs of the whole macular SRCP (*p* = 0.043), parafoveal inferior SRCP (*p* = 0.033), perifoveal SRCP (*p* = 0.048), perifoveal superior SRCP (*p* = 0.027), and perifoveal nasal SRCP (*p* = 0.017) were significantly associated with dementia.

**Table 3:** Associations between selected retinal parameters and cognitive impairment in patients with CSVD.

Retinal parameters	Univariable model		Multivariable model	
	OR (95% CI)	*P*	OR (95% CI)	*P*
Artery mean angle	0.952 (0.916–0.990)	0.013	0.966 (0.927–1.007)	0.104
CRAE/CRVE ratio	0.016 (0.001–0.285)	0.005	0.013 (0.001–0.736)	0.035
Whole macular SRCP	0.834 (0749–0.929)	0.001	0.864 (0.758–0.985)	0.029
Parafoveal SRCP	0.888 (0.821–0.960)	0.003	0.926 (0.848–1.012)	0.089
Parafoveal temporal SRCP	0.887 (0.815–0.966)	0.006	0.929 (0.845–1.021)	0.126
Parafoveal superior SRCP	0.923 (0.868–0.982)	0.012	0.955 (0.891–1.024)	0.193
Parafoveal nasal SRCP	0.915 (0.856–0.977)	0.008	0.953 (0.885–1.027)	0.207
Parafoveal inferior SRCP	0.887 (0.822–0.957)	0.002	0.913 (0.841–0.992)	0.032
Perifoveal SRCP	0.831 (0.747–0.925)	0.001	0.862 (0.755–0.984)	0.027
Perifoveal temporal SRCP	0.883 (0.809–0.964)	0.006	0.921 (0.828–1.025)	0.132
Perifoveal superior SRCP	0.833 (0.753–0.922)	0.000	0.855 (0.755–0.968)	0.014
Perifoveal nasal SRCP	0.799 (0.711–0.897)	0.000	0.833 (0.728–0.952)	0.008
Perifoveal inferior SRCP	0.870 (0.791–0.957)	0.004	0.906 (0.807–1.016)	0.091
Whole macular DRCP	0.881 (0.812–0.956)	0.002	0.948 (0.854–1.052)	0.313
Parafoveal DRCP	0.917 (0.835–1.008)	0.072	1.022 (0.913–1.145)	0.701
Parafoveal temporal DRCP	0.912 (0.828–1.003)	0.058	1.003 (0.900–1.117)	0.963
Parafoveal superior DRCP	0.958 (0.898–1.022)	0.192	1.024 (0.948–1.106)	0.544
Parafoveal nasal DRCP	0.958 (0.886–1.036)	0.283	1.051 (0.947–1.166)	0.351
Parafoveal inferior DRCP	0.894 (0.815–0.981)	0.018	0.965 (0.860–1.082)	0.534
Perifoveal DRCP	0.885 (0.823–0.953)	0.001	0.944 (0.860–1.035)	0.220
Perifoveal temporal DRCP	0.878 (0.807–0.954)	0.002	0.948 (0.857–1.048)	0.296
Perifoveal superior DRCP	0.893 (0.835–0.956)	0.001	0.938 (0.861–1.022)	0.147
Perifoveal nasal DRCP	0.916 (0.861–0.976)	0.006	0.978 (0.903–1.060)	0.585
Perifoveal inferior DRCP	0.897 (0.841–0.956)	0.001	0.943 (0.872–1.102)	0.144
FD-300 area density	0.880 (0.805–0.962)	0.005	0.936 (0.848–1.034)	0.192
Inside-disc RPC	0.939 (0.867–1.017)	0.123	0.916 (0.820–1.023)	0.119
Inside-disc capillary RPC	0.878 (0.806–0.956)	0.003	0.901 (0.837–0.990)	0.028

GCC, ganglion cell complex; RNFL, retinal nerve fibre layer; SRCP, superficial retinal capillary plexus; DRCP, deep retinal capillary plexus; CRVE, central retinal venular equivalent; CRAE, central retinal arteriolar equivalent; FD-300 area density, foveal vessel density in a 300-μm-wide zone around the foveal avascular zone; RPC, radial peripapillary capillary; OR, odds ratio; CI, confidence interval.

### Screening performance of multidimensional retina-related models

**Figure 2:**
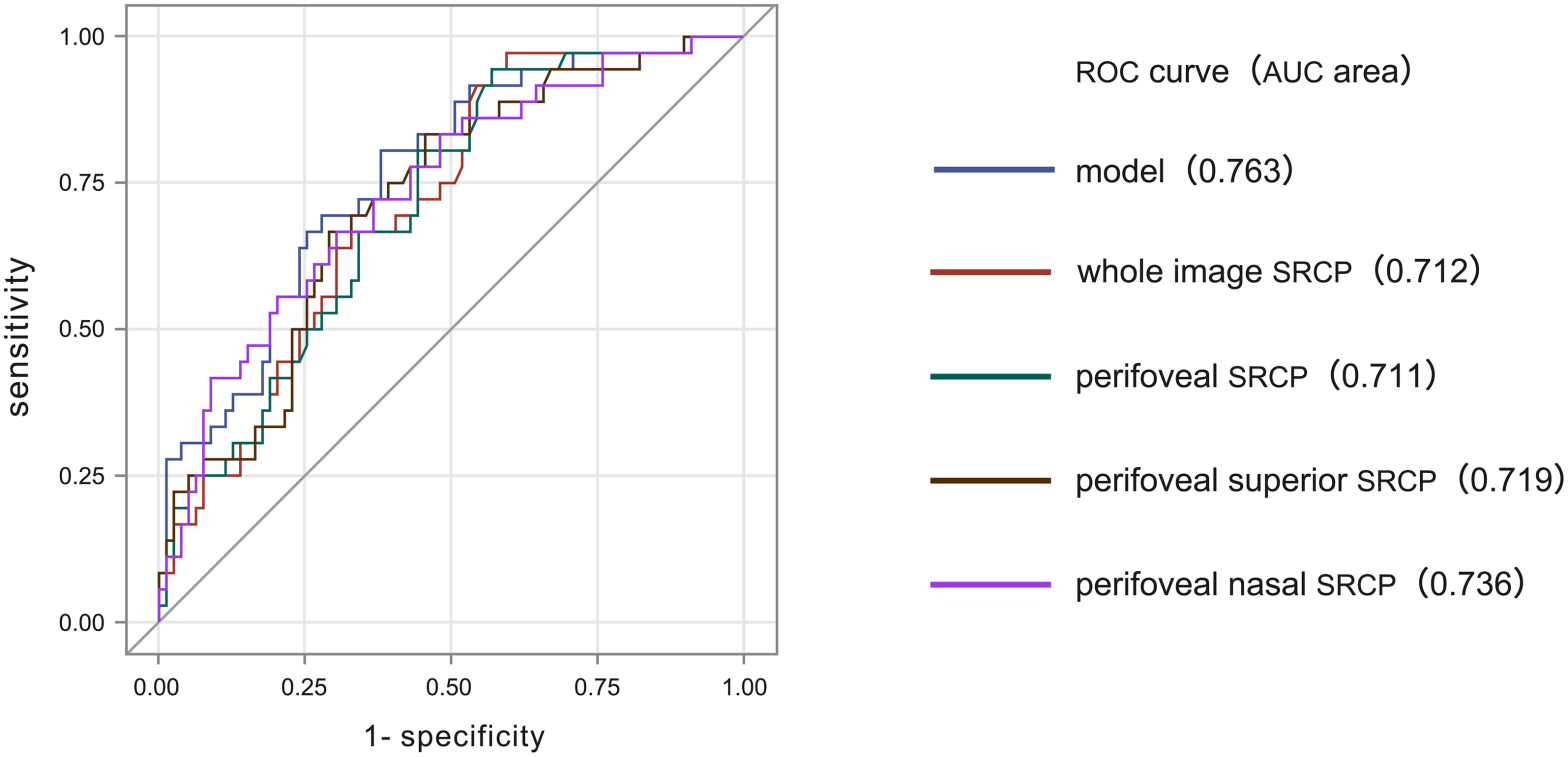
The receiver operating characteristic (ROC) curves of the associations of cognitive impairment with the whole macular superficial retinal capillary plexus (SRCP), perifoveal SRCP, perifoveal superior SRCP, and perifoveal nasal SRCP in patients with cerebral small vessel disease (CSVD). Red line: The area under the ROC curve (AUC) for the vessel density (VD) of the whole macular SRCP was 0.712 (cut-off = 47.81, sensitivity = 97.2%, specificity = 50.5%) for predicting cognitive function *via* the Mini-Mental State Examination (MMSE). Green line: The AUC for the perifoveal SRCP VD was 0.711 (cut-off = 53.37, sensitivity = 94.4%, specificity = 43.1%) for predicting cognitive function *via* the MMSE. Brown line: The AUC for the perifoveal superior SRCP VD was 0.719 (cut-off = 57.25, sensitivity = 83.3%, specificity = 54.4%) for predicting cognitive function *via* the MMSE. Purple line: The AUC for the perifoveal nasal SRCP VD was 0.736 (cut-off = 54.49, sensitivity = 66.7%, specificity = 70.0%) for predicting cognitive function *via* the MMSE.

The screening performance of all multidimensional retina-related models was assessed. Figure 2 presents the ROC curves and corresponding AUCs for the selected four models. The whole macular SRCP VD yielded an AUC of 0.71 (Figure 2, red line) with an optimal cut-off of 47.81, sensitivity of 97.2%, and specificity of 50.5%. The perifoveal SRCP VD also had an AUC of 0.71 (Figure 2, green line) with a cut-off of 53.37, sensitivity of 94.4%, and specificity of 43.1%. The perifoveal superior SRCP VD achieved an AUC of 0.72 (Figure 2, brown line) with a cut-off of 57.25, sensitivity of 83.3%, and specificity of 54.4%. The perifoveal nasal SRCP VD had the highest AUC of 0.74 (Figure 2, purple line) with a cut-off of 54.49, sensitivity of 66.7%, and specificity of 70.0%.

### Associations between retinal features and cognitive domain functions in patients with CSVD

The associations between retinal features and cognitive function of different domains in patients with CSVD are summarised in Table 4. Attention and numeracy scores were significantly correlated with the VDs of the whole macular SRCP (*p* = 0.027), whole macular DRCP (*p* = 0.004), and CRVE (*p* = 0.016), even after adjusting for age, hypertension, diabetes, ischaemic stroke, the Fazekas score of WMHs, and the CSVD burden score. Orientation function was correlated with the VDs of the perifoveal SRCP (*p* = 0.037) and whole-macular DRCP (*p* = 0.044). Visuospatial function (*p* = 0.026) and language competence (*p* = 0.012) were both associated with the inside-disc capillary RPC VD. After adjusting for education, the associations remained significantly different for all variables, excluding language competence and the inside-disc capillary RPC VD. No significant correlations were observed between the memory function score and any retinal parameters.

**Table 4:** Correlations between retinal parameters and different cognitive domains in patients with CSVD.

Retinal parameters	Memory		Visual space		Attention and numeracy		Language competence		Orientation function	
	Beta coefficient (95% CI)	*p*	Beta coefficient (95% CI)	*p*	Beta coefficient (95% CI)	*p*	Beta coefficient (95% CI)	*p*	Beta coefficient (95% CI)	*p*
Whole macular SRCP	0.087 (−0.379–0.554)	0.714	−0.110 (−0.312–0.092)	0.286	0.734 (0.085–1.383)	0.027	0.310 (−0.384–1.004)	0.382	0.264 (−0.487–1.014)	0.492
Perifoveal SRCP	1.821 (−3.692–7.334)	0.517	0.407 (−1.982–2.797)	0.738	−3.495 (−11.162–4.173)	0.372	−4.100 (−12.285–4.102)	0.327	−9.460 (−18.332−−0.588)	0.037
Whole macular DRCP	−0.203 (−0.474–0.069)	0.407	0.035 (−0.082–0.153)	0.557	−0.553 (−0.931−−0.175)	0.004	1.264 (−0.647–0.162)	0.240	−0.449 (−0.886−−0.011)	0.044
Inside-disc capillary RPC	0.034 (−0.002–0.070)	0.060	0.018 (0.002–0.033)	0.026	0.046 (−0.004–0.096)	0.072	0.068 (0.015–0.122)	0.012	0.051 (−0.006–0.109)	0.080
CRVE	−0.008 (−0.017–0.001)	0.063	−0.003 (−0.007–0.000)	0.080	−0.015 (−0.027−−0.003)	0.016	−0.003 (−0.016–0.010)	0.660	−0.004 (−0.018–0.010)	0.569

SRCP, superficial retinal capillary plexus; DRCP, deep retinal capillary plexus; CRVE, central retinal venular equivalent; RPC, radial peripapillary capillary; CI, confidence interval.

## Discussion

In this study, we employed multimodal retinal imaging to investigate the associations between retinal microvascular parameters and cognitive function in patients with CSVD. We observed that a lower CRAE/CRVE ratio and decreased VDs in the macular perifoveal SRCP (especially in the superior and nasal regions) and inside-disc capillary RPC network were associated with lower MMSE scores. Moreover, attention, numeracy, and orientation functions were associated with the VDs of the SRCP and DRCP, whereas visuospatial and language abilities were associated with the VD of the inside-disc RPC network.

To our knowledge, this is the first study to demonstrate that decreased VDs of the superior and nasal perifoveal SRCP and the inside-disc RPC network remained significantly associated with lower MMSE scores after adjusting for age, hypertension, diabetes, ischaemic stroke, white matter hyperintensity severity (Fazekas scores), and the total CSVD burden score. Several mechanisms might explain these findings. Under conditions of mild ischemia and hemodynamic instability, retinal autoregulation initially compensates through the SRCP, making this plexus particularly sensitive to ischemic changes (Wang et al., 2024). The VD of the nasal perifoveal SRCP, which reflects the metabolic status of the papillomacular bundle (Wiseman et al., 2023), might be especially vulnerable because of the high metabolic demand of this structure. Thus, alterations in the retinal microvasculature likely mirror disturbances in cerebral microcirculation (Abdolahi et al., 2024). Our findings are consistent with previous studies demonstrating associations between retinal vascular alterations and cognitive decline (Wang et al., 2021; Huang et al., 2024). Wang et al., (2021) reported that a lower VD of the macular SRCP was associated with worse cognitive function. Retinal vascular abnormalities such as changes in VDs and fractal dimensions, blood flow, and the FAZ (typically enlargement in early-stage AD and mild cognitive impairment) have been reported in patients with cognitive decline (Chiara et al., 2022). Recent evidence further suggests that the nasal macular SRCP VD is associated with hippocampal volume independent of the amyloid status, and lower VD is correlated with subiculum/presubiculum atrophy in cognitive impairment (García-Sánchez et al., 2024). Notably, among the retinal vascular layers, the SRCP has stronger autoregulation capabilities than does the DRCP (Wang et al., 2021). The inside-disc RPC network is composed of only the capillary vasculature within a 2-mm-diameter circle centred on the optic disc (Tao et al., 2022), and it is more sensitive to impaired perfusion of the microvascular system than the remaining RPC network (Serra et al., 2025). Previous studies reported that patients with amnestic mild cognitive impairment exhibited a reduction in the VD of the RPC, both across the entire retinal image and specifically within the peripapillary and inside-disc regions, compared with that in healthy controls (Montorio et al., 2022). The VD of the inside-disc RPC network might be associated with the grey matter volume in patients with AD, potentially influencing cognitive performance (Zhao et al., 2023). Moreover, several studies have demonstrated that better cognition is associated with an increased retinal VD in patients with AD and ischaemic stroke (García-Sánchez et al., 2024; Fu et al., 2022). Collectively, these findings, together with our results, suggest that the SRCP and inside-disc capillary RPC, which represent the VDs in the macula and optic disc, respectively, might serve as sensitive indicators of cognitive impairment severity.

Additionally, we found that attention, numeracy, and orientation functions were correlated with the VDs of the SRCP and DRCP, whereas visuospatial and language abilities were associated with the VD of the inside-disc RPC. These results align with previous findings indicating that retinal capillary perfusion is correlated with specific cognitive domains assessed by MoCA subtasks, including visuospatial and executive functions (e.g., trail making, clock drawing, and cube copying subtasks) (Ashimatey et al., 2020). The VD of the SRCP was reported to be associated with global function, memory function, information processing speed, executive function, and attention function (Wang et al., 2021; Huang et al., 2024).

Moreover, our study revealed that a smaller CRAE/CRVE ratio, reflecting narrower arterioles and wider venules, was associated with poorer cognitive performance. The decrease of the retinal arteriolar diameter reflects structural sclerosis of cerebral small arterioles, endothelial dysfunction, chronic ischaemia, and the cumulative effects of shared risk factors in patients with cerebral small vessel disease. Venular widening is known to indicate reduced arteriolar oxygen saturation, suggesting that increased venular calibre may reflect diminished cerebral oxygen supply (de Jong et al., 2008). This damage significantly impairs cognitive function. This study might be the first to quantitatively explore the associations between morphological changes of the retinal vasculature and cognitive functions in patients with CSVD. Other studies reported associations between arterial parameters or fractal dimensions and cognitive decline in patients with stroke or AD (Jin et al., 2025; Chua et al., 2020). Previous studies demonstrated that retinopathy is associated with subclinical cerebral infarcts and white matter lesions in CSVD (Cheung et al., 2010; Li et al., 2024; Bermudez et al., 2025; Cao et al., 2025; Liew et al., 2014; Ikram et al., 2006). In the present study, we did not observe significant correlations between retinal vascular parameters, including mean angle, angle tortuosity, arc length tortuosity, or fractal dimension, and cognitive impairment. Isolated retinal vascular parameters might not correlate with cognitive impairment because the CRAE/CRVE ratio, which combines arteriolar narrowing (indicating sclerosis) and venular widening (indicating hypoxia/inflammation), better captures microvascular dysfunction and impaired cerebral oxygen delivery linked to cognition. Such structural alterations in the retinal microvasculature reinforce the concept that the retina can serve as a non-invasive window into cerebral microvascular health and potentially provide valuable insights into the vascular mechanisms underlying cognitive dysfunction.

In this study, we did not find associations between retinal structural parameters (i.e., thickness of the GCC or RNFL) and cognitive decline in patients with CSVD. Several reasons could explain these results. Prior studies in AD illustrated that significant GCC thinning is strongly associated with both AD and mild cognitive impairment (Ito et al., 2020; Chua et al., 2020). A study using a murine humanised AD model reported widespread retinal changes across multiple layers, including the RNFL, GCC, outer plexiform layer, and nuclear layers (Sánchez-Puebla et al., 2024). Moreover, pathological analysis of retinas from patients with AD identified amyloid beta plaque deposition within the inner retina, especially in the GCC layers, suggesting that such deposits can trigger inflammation and lead to GCC degeneration (de Jong et al., 2008). However, the retinal structure alternations in CSVD are primarily thought to result from chronic hypoperfusion and impaired retinal oxygenation (Wiseman et al., 2023); therefore, retinal neurodegeneration might occur at more advanced stages of CSVD or cognitive impairment rather than during early stages.

Compared with previous evaluations based on a single parameter, this study provided a more comprehensive assessment by integrating quantitative retinal vascular morphological parameters, OCT-derived retinal structural parameters, and OCTA-derived retinal vascular density. We established several retina-based predictive models to evaluate cognitive function. The selected models, each with defined cut-offs, demonstrated potential utility in the preliminary identification of individuals at risk for dementia. These models can be flexibly applied in clinical practice by adjusting sensitivity and specificity thresholds to meet varying diagnostic needs.

However, our study had several limitations. First, patient recruitment from a specialised neurology centre might limit the generalisability of the findings to community cases, and the lack of correction for multiple comparisons among correlated retinal parameters might have increased the risk of type I errors. Second, although we excluded participants with high refractive error (±6 D) and verified that the included cohort had mild refractive error on average, we did not systematically adjust for ocular covariates such as intraocular pressure, refractive error, or axial length in all models. Uncorrected magnification effects and ocular biometry could introduce bias in quantitative retinal measurements. Third, some models displayed high sensitivity but relatively low specificity, limiting their screening use. Future studies should combine multiple features to improve diagnostic accuracy. Fourth, the MMSE was chosen because it is a brief, widely validated, and clinically practical tool for global cognitive screening, particularly in hospital-based settings in which time and resource constraints exist. However, we acknowledge that the MMSE has limited sensitivity for detecting mild cognitive impairment and specific deficits in executive function. As such, our findings primarily reflect associations with global cognitive impairment. Future studies should incorporate more comprehensive neuropsychological batteries to capture the full spectrum of CSVD-related cognitive dysfunction.

## Conclusion

Overall, our study indicated that the VDs of the macular perifoveal SRCP (especially the perifoveal superior and nasal SRCP) and inside-disc capillary RPC network and the CRAE/CRVE ratio might be indicators of dementia. Furthermore, attention, numeracy, and orientation functions were correlated with the VDs of the SRCP and DRCP, whereas visuospatial and language functions scores might be associated with the VD of the inside-disc RPC. These findings suggest that multimodal retinal evaluation might provide a valuable, disease-specific adjunct for screening cognitive impairment in patients with CSVD.

## Data Availability

The raw data supporting the conclusions of this article will be made available by the authors, without undue reservation.

## Glossary

CSVD: Cerebral small vessel disease
MMSE: Mini-Mental State Examination
VDs: Vessel densities
RPC: Radial peripapillary capillary
SRCP: Superficial retinal capillary plexus
DRCP: Deep retinal capillary plexus
ROC: Receiver operating characteristic
OR: Odds ratio
MoCA: Montreal Cognitive Assessment
OCT: Optical coherence tomography
OCTA: Optical coherence tomography angiography
pRNFL: Peripapillary retinal nerve fibre layer
WMHs: White matter hyperintensities
MRI: Magnetic resonance imaging
STRIVE: Standards for Reporting Vascular Changes on Euroimaging
GCC: Ganglion cell complex
FAZ: Foveal avascular zone
CRAE: Central retinal arteriolar equivalent
CRVE: Central retinal venular equivalent
AUC: Area under the curve
