# Supplementary material for: Associations between multimodal retinal measurements and cognitive functions in patients with cerebral small vessel disease
Source: Front Neurosci. 2026 Mar 20;20:1773266. doi: 10.3389/fnins.2026.1773266 (PMC13047173; doi:10.3389/fnins.2026.1773266)
Supplement: Supplementary file 1 [file Data_Sheet_1.pdf]

Supplementary materials

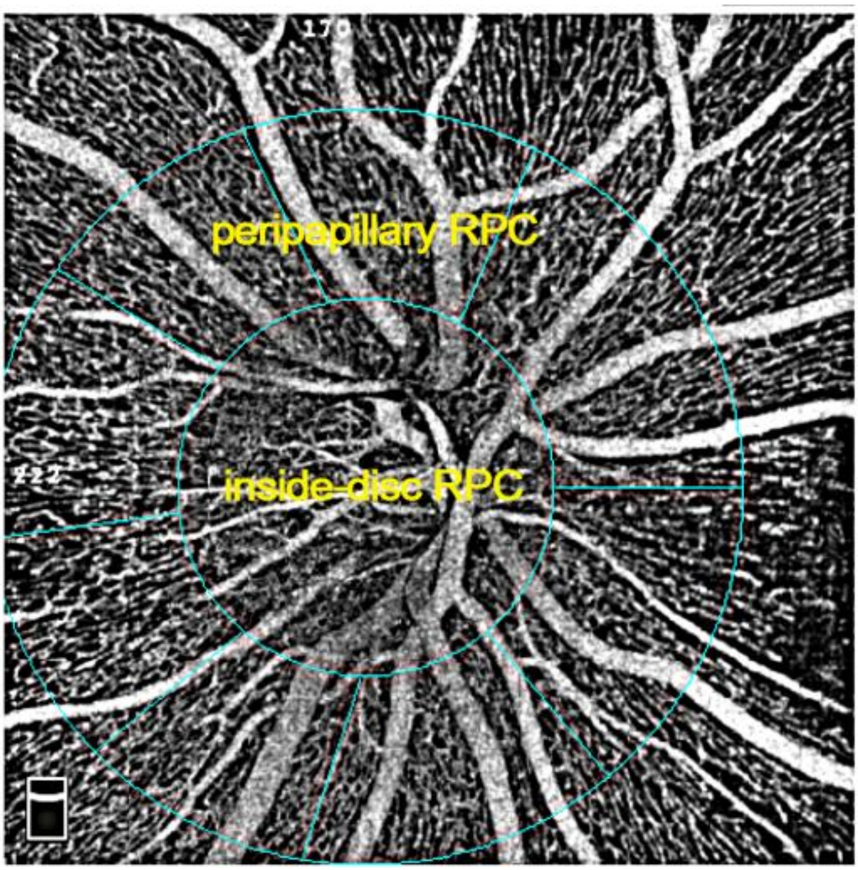

Supplementary Figure 1: Demonstration of optic disc segmentation in optical coherence tomography angiography images.
The radial peripapillary capillary (RPC) network, inside-disc RPC network (the RPCs in an area inside an ellipse fitted to the optic disc boundary), and peripapillary RPC network (the RPCs within a 0.75-mm-wide elliptical annulus extending outwards from the optic disc.

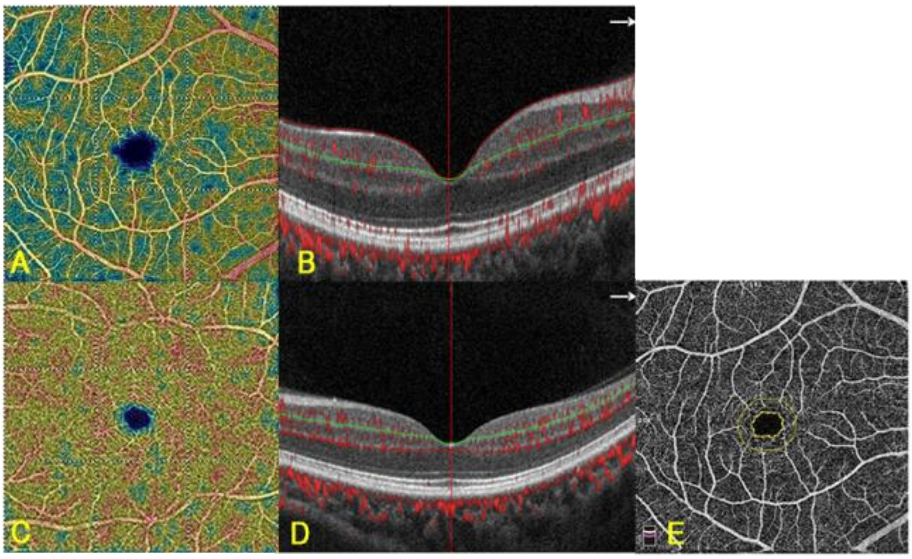

Supplementary Figure 2: Demonstration of macular segmentation in optical coherence tomography angiography image.

A: superficial retinal capillary plexus (SRCP); B: the cross-sectional segmentation of the SRCP; C: deep retinal capillary plexus (DRCP); D: the cross-sectional segmentation of the DRCP; E: foveal avascular zone.

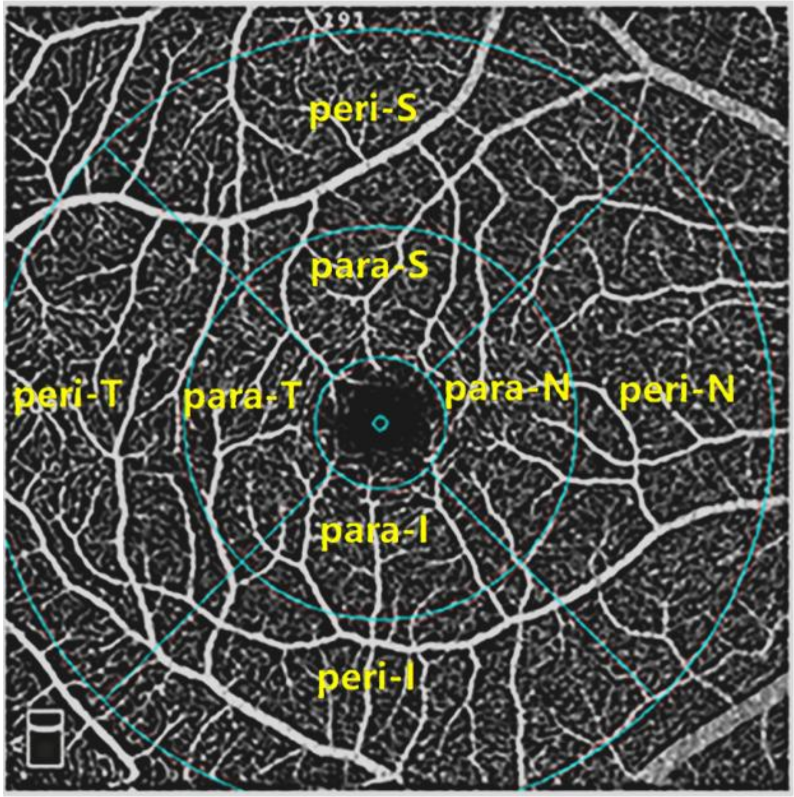

Supplementary Figure 3: Macular (6.0 × 6.0 mm) region was scanned for vessel density measurement.

The macular region was divided into three subfields, namely the foveal (1 mm around the fovea), parafoveal (1–3 mm around the fovea), and perifoveal subfields (3–6 mm around the fovea). Both regions were divided automatically into four subfields: parafovea superior (para-S), parafovea inferior (para-I), parafovea nasal (para-N), and parafovea temporal (para-T), as well as perifovea superior (peri-S), perifovea inferior (peri-I), perifovea nasal (peri-N), and perifovea temporal (peri-T).

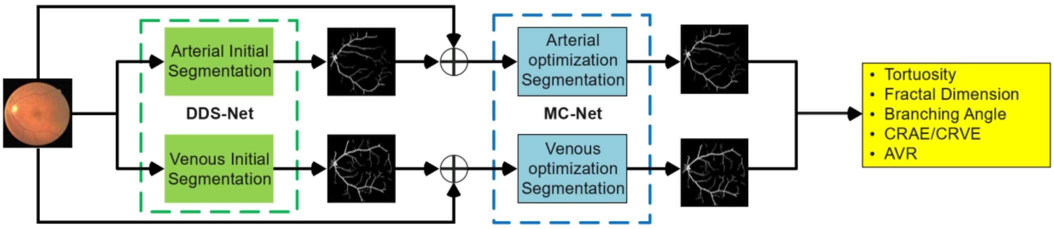

Supplementary Figure 4: Quantitative analysis of retinal blood vessels by a deep learning deep dual-supervised network (DDS-Net) and multicascade network (MC-Net).

We used a fully automatic retinal vessel segmentation and parameter calculation method to calculate the retinal arteriovenous parameters. The constructed DDS-Net was used to extract the deep features from the retinal fundus images for segmenting the retinal arteries and veins, obtaining the initial segmentation map. Then, the constructed MC-Net was used to perform refined vessel segmentation based on the fundus images and initial segmentation map, resulting in a precise segmentation map. Then the retinal arteriovenous parameters were calculated on the basis of the segmented vessels. CRAE, central retinal arteriolar equivalent; CRVE, central retinal venular equivalent. AVR, central retinal arteriolar equivalent/central retinal venular equivalent ratio.

Supplementary Table 1: Inclusion and exclusion criteria

**Inclusion criteria**
1) Age ≥ 18 years.<br>2) Presence of white matter hyperintensities (WMHs) on magnetic resonance imaging: (1) WMHs, Fazekas score ≥ 2; (2) WMHs, Fazekas score = 1 with more than two vascular risk factors, including hypertension, hyperlipidaemia, diabetes, obesity, current smoking, and past medical history of vascular events excluding stroke; and (3) WMHs, Fazekas score = 1 with subcortical lacune.<br>3) Independent in activities of daily living (modified Rankin Scale ≤ 2).<br>4) Signed informed consent form.
**Exclusion criteria**
1) A history of significant ophthalmic diseases, including glaucoma, age-related macular degeneration, diabetic retinopathy, severe cataracts (Lens Opacities Classification System III grade ≥3), retinal vascular occlusions, uveitis, and high myopia (spherical equivalent ≤ −6.0 D)*. A history of intraocular surgery or an inability to maintain a sitting position or fixate steadily for 10–20 s.<br>2) Acute ischemic infarction with maximum diameter > 20 mm on diffusion-weighted imaging/apparent diffusion coefficient.<br>3) Acute haemorrhagic stroke.<br>4) Acute subarachnoid haemorrhage, untreated cerebrovascular malformation, or untreated haemangioma with diameter > 3 mm.<br>5) Definite diagnosis of neurodegenerative diseases, such as Alzheimer's disease and Parkinson's disease.<br>6) Definite diagnosis of white matter hyperintensities of presumed non-vascular origin, such as multiple sclerosis, adult-onset leukodystrophy, and metabolic encephalopathy.<br>7) Definite diagnosis of mental disorder according to the 5th edition of Diagnostic and Statistical Manual of Mental Disorders criteria.<br>8) Contraindications for magnetic resonance imaging, *e.g.*, claustrophobia.<br>9) Severe organic diseases, such as malignant tumour, with life expectancy < 5 years.<br>10) Unable to complete follow-up because of geographic or other reasons.<br>11) Participation in other clinical trials simultaneously.

*Ophthalmic conditions were assessed through a standardised screening protocol conducted by a trained ophthalmologist (W.M.Z) or optometrist at the Department of Ophthalmology, Beijing Tiantan Hospital. The screening included best-corrected visual acuity testing, intraocular pressure measurement, slit-lamp examination, and dilated fundus examination.
